# Predictors of Food and Water Stockpiling During the COVID-19 Pandemic Among Latinos and Non-Latino Black People

**DOI:** 10.1017/dmp.2023.74

**Published:** 2023-07-31

**Authors:** Olufemi Kehinde Fabusoro, Chelsea R Singleton, Margarita Teran-Garcia, Sandraluz Lara-Cinisomo

**Affiliations:** 1 Division of Nutritional Sciences, University of Illinois Urbana-Champaign, Illinois, USA; 2 Department of Social, Behavioral, and Population Sciences, Tulane School of Public Health and Tropical Medicine, New Orleans, USA; 3 Illinois Extension, College of Agricultural, Consumer and Environmental Sciences, University of Illinois Urbana-Champaign, Illinois, USA; 4 Department of Kinesiology and Community Health, University of Illinois Urbana-Champaign, Illinois, USA

**Keywords:** Latinos, Non-Latino Blacks, food and water stockpiling, COVID-19

## Abstract

**Objective:**

The study examined factors associated with food and water stockpiling (FWS) during the COVID-19 pandemic.

**Methods:**

A secondary analysis of online survey data collected in two waves: April 2020 (wave 1) and June/July 2020 (wave 2), was conducted through REDCap web application. A total of 2,271 Non-Latino Black and Latino adults (mean age: 36.8 years (SD = 16.0); 64.3% female) living in Illinois were recruited. Participants self-reported if they stockpiled food and/or water (FWS) seven days prior to survey completion because of the pandemic. Logistic regression was used to determine if each variable was associated with the odds of reporting FWS.

**Results:**

Nearly a quarter (23.3%) of participants reported FWS. The adjusted model revealed that odds of FWS increase as the number of household members increased (OR: 1.21; 95% CI: 1.05-1.41). Odds of FWS were lower among participants who were not self-quarantining compared to those self-quarantining all the time (OR: 0.32; 95% CI: 0.17 – 0.62). Furthermore, individuals with lower levels of concern about COVID-19 had lower odds of FWS than those extremely concerned.

**Conclusions:**

Household size, self-quarantine status, and concern about COVID-19 were significantly associated with FWS. These findings highlight the need to address the concerns of marginalized individuals to promote healthy behaviors.

## Introduction

The Coronavirus Disease (COVID-19) has affected the lives of people and economies worldwide since being declared a global pandemic by the World Health Organization (WHO) in March 2020.^1^ In the early months of the COVID-19 pandemic, many businesses closed temporarily or permanently, and non-essential workers were forced to comply with local and/ or, state stay-at-home orders. As consumers began quarantining at home, they grew concerned about possible food shortages and price gouging. As a result, consumers across the world started stockpiling food and water.^2^

The economic fallout of the COVID-19 pandemic has disproportionately affected racial and ethnic minorities in the US. For example, Black and Latino adults have experienced higher rates of pandemic-related job loss. They have a higher risk of contracting the disease because they are more likely to hold essential jobs (e.g., food retail, public transportation, etc.), making it difficult to quarantine and avoid exposure.^3,4^ Although there are studies of stockpiling behavior and preferences related to COVID-19,^5^ it is unclear how food and water stockpiling (FWS) behaviors are associated with pandemic-related factors, such as job loss and quarantining behaviors among marginalized groups (i.e., Black people and Latinos) in the US. Therefore, we sought to identify individual, household, and COVID-19 related factors associated with FWS early in the pandemic among non-Latino Black people and Latino adults living in Illinois, to identify potentially vulnerable individuals in future large-scale health crises.

## Method

Self-administered survey data was collected through the Research Electronic Data Capture web application to measure the early effects of the COVID-19 pandemic among adults (aged ≥ 18 years) residing in Illinois. Surveys were collected in April, 2020 (n = 2294) and June/ July, 2020 (n = 2143). For the current study, only Latino and non-Latino Black participants were included (n = 2271, 51% of the total sample). The Institutional Review Board at the University of Illinois Urbana-Champaign (protocol number: 20868) approved this research.

This secondary data analysis included participants’ self-reported FWS behavior, sociodemographic and household characteristics, anxiety symptoms, and Supplemental Nutrition Assistance Program (SNAP) participation, as well as self-quarantine behaviors, concern about COVID-19, and job changes due to the pandemic. Self-reported household food security status was measured using the US Department of Agriculture’s 6-item food security module.^6^ Anxiety symptoms were assessed using the validated, 2-item Generalized Anxiety Disorder-2 (GAD-2) scale.^7^

Descriptive statistics (i.e., means and frequencies) were calculated and stratified by FWS status. Logistic regressions tested the association between individual, household, and COVID-19 related variables, and the odds of reporting FWS. Variables that were statistically significant in the crude models were included in the adjusted model. Statistical significance was assessed at α = 0.05. All analyses were performed with SAS version 9.4 (SAS Institute Inc., Cary, North Carolina, USA).

## Results

Table 1 shows the sample characteristics and results from the crude and adjusted logistic regression models. About a quarter of the participants reported FWS. The adjusted logistic regression revealed that the odds of FWS increased as the number of household members increased (OR = 1.21, 95% CI [1.05 - 1.41]). There was a dose-response relationship between self-quarantining status and odds of FWS. The odds of FWS decreased as the frequency of quarantining decreased. Participants who endorsed feeling ‘a little’ or ‘moderately’ concerned about COVID-19 had lower odds of FWS compared with those extremely concerned (OR = 0.60, 95% CI [0.36 - 0.99], and OR = 0.64, 95% CI [0.46 - 0.91], respectively). Individual characteristics, other household variables (e.g., SNAP participation and food security status), anxiety, and job changes due to the pandemic were not significantly associated with FWS in the adjusted model.

**Table 1.** Associations between individual, household, anxiety, and pandemic-related measures and food and water stockpiling during the COVID-19 pandemic

Measure:	All Participants	Reported Stockpiling	Did Not Report Stockpiling	Crude Models	Adjusted Model
	n = 2271	530 (23.3)	1741 (76.7)	OR (95% CI)^a^	OR (95% CI)^b^
*Individual*					
Age (years), mean (±sd)	36.8 (±16.0)	36.8 (±15.1)	36.8 (±16.3)	1.00 (0.99 – 1.01)	–
Sex, n (%)					
Female	1439 (64.3)	367 (69.4)	1072 (62.7)	**1.35 (1.10 – 1.66)**	1.00 (0.99 – 1.01)
Other^c^	800 (35.7)	162 (30.6)	638 (37.3)	REF	REF
Race/Ethnicity, n (%)					
Latino	856 (37.7)	240 (45.3)	616 (35.4)	**1.51 (1.24 – 1.84)**	1.05 (0.85 – 1.44)
Non-Latino Black	1415 (62.3)	290 (54.7)	1125 (64.6)	REF	REF
Education Level, n (%)					
≤ HS Diploma/ GED	672 (30.0)	145 (27.4)	527 (30.8)	REF	REF
Some College/ Vocational	733 (32.7)	177 (33.5)	556 (32.5)	1.16 (0.90 – 1.49)	–
College Degree	565 (25.2)	135 (25.5)	430 (25.1)	1.14 (0.87 – 1.49)	–
Graduate Degree	272 (12.1)	72 (13.6)	200 (11.7)	1.31 (0.94 – 1.81)	–
Annual Income (Pre-Pandemic), n (%)					
< $20,000	615 (29.2)	115 (23.1)	500 (31.1)	REF	REF
$20000 - $49999	650 (30.9)	160 (32.1)	490 (30.5)	**1.42 (1.08 – 1.86)**	1.15 (0.82 – 1.63)
$50,000 - $99999	565 (26.8)	156 (31.3)	409 (25.4)	**1.66 (1.26 – 2.18)**	1.42 (0.99 – 2.03)
≥ $100,000	277 (13.2)	68 (13.6)	209 (13.0)	**1.41 (1.01 – 1.99)**	1.24 (0.78 – 1.97)
*Household*					
Number of Household Members, mean (± sd)	2.43 (1.01)	2.55 (0.97)	2.39 (1.01)	**1.17 (1.06 – 1.29)**	**1.21 (1.05 – 1.41)**
Family Structure, n (%)					
No Spouse, No Children	1058 (46.6)	232 (43.8)	826 (47.4)	REF	REF
No Spouse, Children	412 (18.1)	101 (19.1)	311 (17.9)	1.16 (0.89 – 1.51)	1.00 (0.70 – 1.44)
Spouse, No Children	441 (19.4)	99 (18.7)	342 (19.6)	1.03 (0.79 – 1.35)	1.04 (0.74 – 1.47)
Spouse, Children	360 (15.9)	98 (18.5)	262 (15.1)	**1.33 (1.01 – 1.75)**	0.78 (0.52 – 1.16)
SNAP Participation, n (%)					
Yes	570 (25.1)	144 (27.2)	426 (24.5)	1.15 (0.92 – 1.44)	–
No	1701 (74.9)	386 (72.8)	1315 (75.5)	REF	REF
Food Security Status, n (%)					
Food Secure/ Marginal	896 (48.5)	247 (51.0)	649 (47.6)	REF	REF
Low Food Security	646 (35.0)	147 (30.4)	499 (36.6)	**0.77 (0.61 – 0.98)**	0.84 (0.62 – 1.13)
Very Low Food Security	305 (16.5)	90 (18.6)	215 (15.8)	1.10 (0.83 – 1.47)	1.12 (0.79 – 1.59)
*Mental Health*					
Anxiety,^d^ mean (± sd)	1.9 (± 1.9)	1.9 (± 1.9)	1.8 (± 1.9)	1.02 (0.97 – 1.08)	–
*Pandemic*					
Self-Quarantine status, n (%)					
All of the time	528 (25.5)	165 (31.1)	363 (23.5)	REF	REF
Most of the time	873 (42.1)	240 (45.3)	633 (41.0)	**0.83 (0.66 – 1.06)**	**0.71 (0.53 – 0.95)**
Some of the time	501 (24.1)	96 (18.1)	405 (26.2)	**0.52 (0.39 – 0.70)**	**0.52 (0.37 – 0.75)**
None of the time	173 (8.3)	29 (5.5)	144 (9.3)	**0.44 (0.29 – 0.69)**	**0.32 (0.17 – 0.62)**
Concerned about COVID-19, n (%)					
Not at all concerned	108 (5.4)	12 (2.4)	96 (6.4)	**0.26 (0.14 – 0.49)**	0.69 (0.35 – 1.36)
A little concerned	163 (8.1)	29 (5.7)	134 (8.9)	**0.46 (0.30 – 0.70)**	**0.60 (0.36 – 0.99)**
Moderately concerned	496 (24.6)	103 (20.3)	393 (26.1)	**0.55 (0.42 – 0.72)**	**0.64 (0.46 – 0.91)**
Very concerned	600 (29.8)	155 (30.5)	445 (29.5)	**0.73 (0.57 – 0.94)**	0.79 (0.58 – 1.08)
Extremely concerned	648 (29.8)	209 (41.1)	439 (29.1)	REF	REF
Job status change due to pandemic, n (%)					
Lost job entirely	284 (15.6)	85 (17.7)	199 (14.8)	1.25 (0.93 – 1.67)	–
Paid hours reduced	376 (20.6)	113 (23.5)	263 (19.6)	1.25 (0.96 – 1.63)	–
Anticipate job lost	258 (14.1)	50 (10.4)	208 (15.5)	0.70 (0.50 – 0.99)	–
No change	908 (49.7)	232 (48.3)	676 (50.2)	REF	REF
Survey Wave, n (%)^e^					
Wave 1	659 (29.0)	144 (27.2)	515 (29.6)	0.89 (0.72 – 1.10)	–
Wave 2	1612 (71.0)	386 (72.8)	1,226 (70.4)	REF	–

CI, Confidence Interval; HS, High School; OR, Odds Ratio; REF, Reference Group; SNAP, Supplemental Nutrition Assistance Program. Frequencies in cells may not sum to total sample size due to missing data.

a Crude logistic regression models include only the variable as it *affects the outcome (FWS), ignoring potential covariates*. Bold text indicates statistical significance.

b The adjusted logistic regression model includes variables that were statistically significant in the crude models that were adjusted to account for other predictor variables.

c Other includes survey participants who identify as male or non-binary.

d Anxiety measured with the Generalized Anxiety Disorder-2. Scores range from 0 to 6.

e Survey wave reflects the survey completion period: wave 1 in April 2020 and wave 2 in June/July 2020.

There was an upsurge in food stockpiling behavior in countries such as China,^2^ and other European and Asian countries,^8^ after the WHO declaration of COVID-19 as a pandemic. The findings from this study showed that nearly a quarter of non-Latino Black people and Latinos reported FWS. The results also showed that household and pandemic-related factors were associated with FWS. The results indicated that a higher number of family members in the household increased the odds of FWS in this diverse sample with varied levels of education, pre-pandemic annual income, levels of food insecurity, and pandemic-related job changes. These findings likely reflect the need to ensure that every household member had sufficient food and water. Individuals with relatively lower levels of concern about COVID-19 had lower odds of FWS compared to those who endorsed feeling ‘extremely concerned’ about the pandemic. These results support other reports that American, Canadian, and European adults who feared more severe pandemic threats were more likely to stockpile essentials during COVID-19 lockdowns.^9^ Similarly, less frequent quarantining significantly lowered the odds of FWS. It is possible that individuals who were willing to leave their homes during the lockdowns felt more confident about accessing food and water than others in our sample.

## Conclusions

Given the findings, it is imperative that researchers, policymakers, and practitioners further examine FWS behavior and how pandemic-related fear can lead to FWS. Stockpiling may negatively impact supply chains and cause shortages that would be dangerous for vulnerable or marginalized groups due to food inaccessibility. Stockpiling of perishable foods may lead to food that is no longer safe (sub-optimal) in terms of nutrient content, which may compromise nutrition security.^10^ These findings can inform clinicians and healthcare professionals about effective strategies to minimize COVID-19 adverse outcomes for these marginalized groups now and in post-COVID-19 safety efforts. However, future studies should further examine the important variables reported here to inform initiatives that meet marginalized communities’ food and water needs during a large-scale crisis.

## References

[1] World Health Organization. *WHO Director-General’s opening remarks at the media briefing on COVID-19, March 11, 2020*. Geneva, Switzerland; 2020. https://www.who.int/director-general/speeches/detail/who-director-general-s-opening-remarks-at-the-media-briefing-on-covid-19---11-march-2020. Accessed June 28, 2022.

[2] Wang E , An N , Gao Z , Kiprop E , Geng X. Consumer food stockpiling behavior and willingness to pay for food reserves in COVID-19. Food Sec. 2020;12(4):739-747. doi: 10.1007/s12571-020-01092-1 PMC7406878 32837661

[3] Cyrus E , Clarke R , Hadley D , et al. The impact of COVID-19 on African American communities in the United States. *medRxiv*. Published online May 19, 2020. doi:10.1101/2020.05.15.20096552 10.1089/heq.2020.0030 PMC7702977 33269331

[4] Zamarripa R , Roque L. Latinos face disproportionate health and economic impacts from COVID-19. Published 2021. https://www.americanprogress.org/issues/economy/reports/2021/03/05/496733/latinos-face-disproportionate-health-economic-impacts-covid-19/. Accessed July 28, 2021.

[5] Amaral NB , Chang B , Burns R. Understanding consumer stockpiling: insights provided during the COVID-19 pandemic. J Consumer Affairs. 2022;56(1):211-236. doi: 10.1111/joca.12434

[6] United States Department of Agriculture (USDA). *USDA ERS - survey tools. Six-item short form of the food security survey module.* Published 2023. https://www.ers.usda.gov/topics/food-nutrition-assistance/food-security-in-the-u-s/survey-tools/#six. Accessed March 7, 2023.

[7] Plummer F , Manea L , Trepel D , McMillan D. Screening for anxiety disorders with the GAD-7 and GAD-2: a systematic review and diagnostic metaanalysis. Gen Hosp Psychiatry. 2016;39:24-31. doi: 10.1016/j.genhosppsych.2015.11.005 26719105

[8] Ahmadi I , Habel J , Jia M , Lee N , Wei S. Consumer stockpiling across cultures during the COVID-19 pandemic. J Int Marketing. 2022;30(2):28-37. doi: 10.1177/1069031X211037590

[9] Garbe L , Rau R , Toppe T. Influence of perceived threat of COVID-19 and HEXACO personality traits on toilet paper stockpiling. PLoS One. 2020;15(6):e0234232. 32530911 10.1371/journal.pone.0234232 PMC7292383

[10] Pravst I , Chang BPI , Zmitek K. The effects of the COVID-19 outbreak on food supply, dietary patterns, nutrition, and health. Frontiers in Nutrition. 2022;2. doi: 10.3389/978-2-83250-764-3 PMC8905438 35284469

